# Psychosocial determinants of contraceptive desire and use among sexually-active adolescent girls in Kenya and Nigeria: implications for girl-centered contraceptive programs

**DOI:** 10.1186/s40834-025-00416-w

**Published:** 2025-12-08

**Authors:** Abednego Musau, Sophie Schlingemann, Roselyn Odeh, Lydiah Ndungu, Alhassan Bulama, Harmon Momanyi, Meghan Cutherell, Albert Tele, Regien Biesma, Jelle Stekelenburg

**Affiliations:** 1 Population Services International, Nairobi, Kenya; 2 Department of Health Sciences, Global Health Unit, University Medical Centre Groningen, Groningen, Netherlands; 3 Society for Family Health Nigeria, Abuja, Nigeria; 4 Population Services Kenya, Nairobi, Kenya; 5 Population Services International, Washington, DC USA; 6 Vrije Universiteit, Amsterdam, Netherlands; 7 Julius Center Global Health, University Medical Center Utrecht, Utrecht, Netherlands; 8 Department Obstetrics & Gynaecology, Frisius Medical Center, Leeuwarden, Netherlands

**Keywords:** Sexually-active adolescent girls, Psychosocial determinants, Desire for contraception, Current contraceptive use, Preference-aligned contraceptive use, Sexual and reproductive health

## Abstract

**Introduction:**

The high rates of unintended and early pregnancies among adolescent girls in sub-Saharan Africa are concerning and are substantially contributed to by low contraceptive uptake. Contraceptive use can avert these pregnancies, but demand remains low. We investigated the influence of psychosocial determinants on three outcomes: desire for contraception, current use and preference-aligned use among sexually-active adolescent girls from Kenya and Nigeria.

**Methods:**

Our study involved data from household-based cross-sectional surveys in 282 primary sampling units in two Nigerian states and four Kenyan counties. Participants were adolescent girls aged 15–19 years. The data was collected after mapping and listing of the households where eligible participants resided. Six psychosocial determinants (contraceptive knowledge, perceived self-efficacy, contraceptive relevance and reproductive control, future aspirations and descriptive norms) were fitted into generalized linear mixed-effects models against the study outcomes.

**Results:**

Data from 2,327 participants were analyzed. The majority (81.5%) were married or living as married, 16.2% were attending school and 60% were Muslim. Three-quarters had ever given birth, 71% had sex weekly while 93.5% had desire for future conception. Overall, 46.0% expressed current desire for contraception, 37.2% were using contraception while 86.5% practiced preference-aligned contraceptive use. Controlling for selected covariates, contraceptive knowledge, perceived contraceptive relevance, self-efficacy and descriptive norms were positively associated with desire for and current use. Future aspirations and perceived reproductive control were not associated with all three outcomes. In the main analysis, no psychosocial determinant was associated with preference-aligned contraceptive use.

**Conclusions:**

Our study demonstrated modest desire for contraception and low contraceptive prevalence amidst high desired fertility, highlighting a critical opportunity for contextualized girl-centered programs. Such programs should focus on enhancing contraceptive knowledge, building self-efficacy, increasing perceived relevance and shifting descriptive norms to drive contraceptive demand and use. Investigated determinants could serve as intermediate outcomes tracked to monitor progress towards contraceptive use outcomes and to inform program adaptations. Despite the high preference-aligned contraceptive use demonstrated, this metric is nascent, and more research is warranted to establish its utility for evaluating programs.

**Supplementary Information:**

The online version contains supplementary material available at 10.1186/s40834-025-00416-w.

## Introduction

Annually, approximately 21 million adolescent pregnancies occur in low- and middle-income countries (LMICs), half of which are unintended [1]. In 2024, Sub-Saharan Africa (SSA) reported the highest adolescent fertility rates globally, ranging from 92 to 101 births per 1,000 girls, two times the global average [2]. Pregnancy in adolescence occurs during a critical phase that bridges childhood to adulthood and is often associated with detrimental health and socioeconomic consequences, including poor maternal, newborn and child health outcomes [3–5], lower education attainment and limited job opportunities, which perpetuate poverty cycles [6, 7]. Contraception can effectively mitigate these socioeconomic challenges, empowering girls to achieve their life aspirations and fostering gender equality [1]. However, contraceptive use among adolescent girls in SSA remains low [8, 9]. Contraceptive decision-making and use among adolescent girls is influenced by many individual, societal and structural factors including socio-cultural norms in the contexts where they live [10, 11]. Changing socio-cultural norms is a complex and resource-consuming process [12]. Contrastingly, individual psychological factors are relatively easily modifiable, offering huge opportunities for programs that seek to enhance decision-making and contraceptive use [13].

For programs to effectively support contraceptive use among adolescent girls from diverse contexts, an understanding of the social and psychological factors that affect desired and actual contraceptive use behavior is essential. Besides, enriching programs with components that address individual’s beliefs, ideas and feelings about fertility and contraception, referred to as ‘ideation or psychological factors’, and social norms could influence girls’ preferences and improve contraceptive decision making. This argument was advanced in the Ideation Model of Strategic Communication with Behavioral Change by Kincaid [14]. The model has been trialed as an analytic framework for establishing determinants of contraceptive use in SSA [15–17]. Ideation factors for contraceptive decision making and use can be grouped into three domains. The cognitive domain includes contraceptive knowledge and contraceptive relevance - a perception that contraceptives are relevant when pursuing one’s life goals. The emotional domain includes perceived contraceptive self-efficacy and one’s confidence to pursue their future aspirations while the social domain entails perceived reproductive control and descriptive norms. Ideation factors intersect with social-cultural norms to influence contraceptive decision making and behaviors.

Understanding the intersection of ideation factors and social constructs in influencing contraceptive behaviors is important as it could inform the design of adolescent-responsive programs. Further, the evidence yielded could facilitate the establishment of reliable measures for assessing the progress made by programs towards improving contraceptive behavioral outcomes. There is a lot of research describing contraceptive use patterns and socio-demographic determinants among adolescent girls, but little is known about the influence of psychosocial determinants on contraceptive preferences and use among adolescent girls in SSA where adolescent fertility rates are highest [8, 9, 18]. Besides, research has paid little attention to individuals’ self-defined preferences especially within contexts where pervasive gender norms obfuscate girls’ decision-making autonomy [11]. The emergence of person-centered contraceptive use measures such as preference-aligned fertility management (PFM) [19, 20] expands the opportunities for understanding how multiple factors could be influencing contraceptive use behaviors. PFM is a measure of the concordance between women’s expressed desire to use contraception and the self-reported contraceptive use status at a particular time [19, 20]. In this era of person-centered approaches, understanding how psychosocial determinants shape contraceptive use behaviors which are well aligned with the unique preferences and specific needs of adolescent girls is important. Our study aimed to establish the influence of psychosocial variables on contraceptive desire and use among sexually-active adolescent girls in Kenya and Nigeria. In addition, we explored how psychosocial determinants influenced preference-aligned contraceptive use.

## Materials and methods

### Study design

Our study was a secondary analysis of pooled data from two cross-sectional surveys conducted in Nigeria and Kenya between October and December 2022. The primary studies were pre-post quasi-experimental studies with pre-intervention and post-intervention phases independently designed to determine the effectiveness of the Adolescents 360 (A360) project’s interventions in Kenya [21] and Nigeria [22]. Briefly, A360 is an adolescent sexual and reproductive health (ASRH) project implemented in Kenya, Tanzania, Nigeria and Ethiopia since 2016. A360 employs a multi-disciplinary approach incorporating human-centered design to evolve context-specific and girl-centered interventions that position contraception as a tool enabling girls to pursue their life aspirations [23]. Our study employed data only from the baseline phase of the pre and post-intervention studies focusing on the A360 project geographies in Nigeria and Kenya. Nigeria and Kenya have poor ASRH indicators underscoring their selection for the A360 program and for our study. From demographic health surveys conducted in 2022 (Kenya) and 2023–2024 (Nigeria), 15% of adolescents 15–19 years from the two countries have ever been pregnant, only 4.3% of Nigerian married girls 15–19 years were using contraception while in Kenya 63% of girls had wanted to delay their most recent pregnancy [24, 25].

In Nigeria, the primary study was conducted in four randomly sampled local government areas (LGAs) in Kaduna (Igabi and Sabon Gari - intervention; Kauru and Sanga - comparison) and in two LGAs in Nasarawa (Doma - intervention and Nasarawa - comparison). The three intervention LGAs were randomly sampled from a line list of all LGAs implementing the A360 intervention in the two states. Comparison LGAs were randomly selected from a list of all non-implementing LGAs in each state excluding LGAs experiencing security challenges. The primary sampling unit (PSU) in Nigeria was an enumeration area (EA) within the selected LGAs. The investigators initially mapped out and line listed all the EAs within a five-kilometer radius from selected health facilities where adolescent girls were receiving contraceptive counselling and services. Thereafter, 182 EAs (45 EAs from Nasarawa and 137 EAs from Kaduna) were randomly selected for the study. In Kenya, the four counties (Kilifi, Narok, Homa Bay and Migori) where A360 was being implemented were all selected as were all the sub-counties in them. The PSU in Kenya was a community health unit (CHU) as defined by the Kenya Community Health Strategy 2020–2025 [26]. Overall, 100 CHUs were randomly selected from all CHUs affiliated to the health facilities where A360 was being implemented: 42 in Migori, 31 in Homabay, 15 in Kilifi and 12 in Narok. EAs in Nigeria and CHUs in Kenya are comparable because they geographically represent around 100 households. In both countries, the estimated samples were allocated to the states or counties proportionate to the target projections of the number of new contraceptive adopters to be reached by the A360 project by June 2025.

### Study population

Aligning with A360’s target beneficiaries, the primary study in Nigeria exclusively targeted married adolescent girls or those living as if married. In Kenya, adolescent girls aged 18–19 years or mature minors (for those aged 15–17 years) were involved as defined by the 2015 Guidelines for Conducting Adolescent HIV and Sexual and Reproductive Health Research in Kenya [27]. In Nigeria, adolescent girls 15–19 were ineligible if they were not married while in Kenya girls aged 15–17 years were ineligible if they were not emancipated. This article only focuses on study records from the sexually-active girls because the dependent outcomes investigated are irrelevant for girls who are not sexually-active.

### Sample size and sampling

The estimated total sample for the primary studies in both geographies was 3,172; 2,183 and 989 participants in Nigeria and Kenya respectively. These sample sizes were estimated for each of the geographies with different estimation assumptions. The sample for Nigeria was estimated to be sufficient to detect an 8%-point difference-in-difference on the proportion of eligible adolescent girls who had used a contraceptive method at the last sexual encounter between the baseline and endline timepoints, comparing intervention and comparison geographies. In Nigeria, a comparison group was involved which doubled the sample size. For Kenya, the primary study was powered to detect a 10% difference in the proportion of eligible adolescent girls who affirmed that contraceptives were relevant to enable them to pursue their life goals between baseline and endline without a comparison group. In both regions, the sample sizes were adjusted to account for clustering using an intracluster correlation coefficient of 0.045 and a 10% non-response rate, ensuring a 95% confidence level and a 5% margin of error. The desired sample in Nigeria was attained, while response rate for Kenya was 97.8%. Consequently 3,150 records were initially available for analysis. However, study records from 502 participants who were pregnant at the time of the survey and 321 participants who had not engaged in sexual activity in the 12 months preceding the survey were excluded from the analysis.

### Recruitment and data collection procedures

The primary studies which yielded the analyzed data were executed in two steps. The first step encompassed the mapping and listing of households where potentially eligible adolescent girls resided. This step was conducted by trained study personnel, accompanied by community workers (community health promoters in Kenya and community elders in Nigeria). By visiting all households mapped within the PSUs, a household mapping screening tool was administered to the senior member of the household during the visit, upon receipt of verbal consent. Personnel assigned each household a unique number and captured the household name, geolocation data, number of adolescent girls aged 15–19 years, and the marital and parity status for each adolescent girl. After completion of this exercise, all the households where potentially eligible participants lived in each PSU were line listed. For each PSU, systematic sampling was employed to select 15 households from the line list aiming to recruit 10–12 participants.

For the second step, trained female enumerators visited the sampled households aided by the geolocation data or physical tracing guided by the community workers. Upon arrival, the pair introduced themselves to the household head or the most senior member of the household and sought permission to speak to the potential participants in the sampled list in a private spot at the household ensuring that sexual and reproductive health were not mentioned in the conversation. Privately with each potentially eligible adolescent girl, the enumerators fielded screening questions inquiring about age, emancipation status and marital status to confirm their eligibility. Once eligibility was confirmed, enumerators performed consenting procedures and fielded a survey questionnaire. The interview was conducted observing auditory and visual privacy and using the offline mode of the android versions of SurveyCTO (Kenya) or CS Pro (Nigeria) applications hosted in institutional electronic devices. The enumerators read the questions as they were written on the questionnaire using English or the preferred local language - either Masai, Dholuo or Swahili in Kenya and Hausa in Nigeria. In a household where two or more eligible participants were found, one was recruited through a random selection process involving folded pieces of paper with one written “yes” and the rest “no” that were provided to the eligible girls for selection. Enumerators moved from one selected household to the other until the desired sample size was attained. The collected data was synced to an institutional server several times daily through internet connection.

### Study measures

Questionnaires for the primary studies gathered three categories of variables: the participants’ sociodemographic characteristics, measures for psychosocial determinants and the dependent variables.

#### Socio-demographic characteristics, fertility and pregnancy

These were participants’ age, marital status, number of children born, whether currently in school or not, religion, sexual frequency, desire for and timing for future pregnancy, and current pregnancy status.

#### Psychosocial determinants

Six variables served as psychosocial determinants: contraceptive knowledge, perceived relevance of contraceptives, contraceptive self-efficacy, future aspirations, perceived reproductive control and descriptive norms. These determinants were measured using a composite of several questions or validated scales used in various studies [28–31] as summarized in Table 7 (Supplementary Material 1).

#### Dependent variables

The dependent variables were: (1) current desire to use contraception, (2) current contraceptive use and (3) preference-aligned contraceptive use. Current desire to use contraception was assessed using the first question among the items designed to measure PFM by Holt et al., [19]. Current contraceptive use was assessed by asking, ‘*Are you or your partner currently doing something or using any method to delay or avoid getting pregnant?*’ with ‘*yes*’ and ‘*no*’ as response options. Participants who responded with a ‘*yes*’ were asked to state the method they were using. Preference-aligned contraceptive use was a dichotomous composite variable generated from the responses on the questions on current desire and current contraceptive use using a modification to the approach by Holt et al., [19]. Preference-aligned contraceptive use was defined as the concordance between the reported current desire to use and actual contraceptive use. Participants responding with “*don’t know*” about their current desire were classified as performing preference-aligned contraceptive use, if they were using a method.

### Data analysis

Collected data was cleaned separately for the Kenya and Nigeria studies and merged into one dataset excluding records from pregnancy participants and those reporting no sexual activity in recent 12 months from the final sample. Frequencies were generated to summarize categorical socio-demographic characteristics, dependent variables, and psychosocial determinants. Means and measures of dispersion were generated to summarize the numeric scores for the psychosocial determinants, which demonstrated good internal consistency as illustrated in Table 8 (Supplementary Material 2). To model how psychosocial determinants influenced the three dependent variables, psychosocial determinants were grouped into three domains i.e., cognitive (contraceptive knowledge and perceived relevance), emotional (self-efficacy and future aspirations) and social (perceived reproductive control and descriptive norms). We fitted multivariable generalized linear mixed effects models (GLMM) with the psychosocial determinants in each domain as independent variables against each of the three dependent variables controlling for age, schooling status, marital status, parity and religion (step 1). The covariates were selected referencing a recent systematic review, which featured Kenya and Nigeria, on the factors associated with contraceptive use among sexually-active adolescent girls in SSA [8]. Our study employed GLMM for analysis because our dependent outcomes were categorical and clustering of data based on the geographic clusters was anticipated. GLMM is a promising approach for analyzing non-normal data, as an extension of generalized linear models, and for handling data which is correlated or where variability is not constant and random effects are involved (as is done through linear mixed effects models) [32]. Our models were fitted using the binomial distribution with a logit link function because of our binary categorical outcomes and robust standard errors employed using Stata 15.0. In step 2, all psychosocial determinants were fitted in one model to examine how they behaved when pooled together using the GLMM approach. Results are tabulated illustrating the adjusted odds ratios (aORs), their 95% confidence intervals (95% CI) and the *p* values. Statistical significance was set apriori to at the 0.05 level.

## Results

### Sample characteristics

Table 1 summarizes the participants’ socio-demographic, sexuality and fertility characteristics. In brief, 1748 (75.1%) were from Nigeria while 579 (24.9%) were from Kenya. The majority (1897, 81.5%) of the respondents were either married or living together as married. Only 376 (16.2%) respondents were attending school. In terms of religious affiliation, 1407 (60%) identified as Muslim, while 796 (34.2%) identified as Protestant. Three out of four respondents had ever given birth, and most of them had given birth once or twice. Besides, 1655 (71.1%) reported sexual activity at least once a week. Importantly, 2174 (93.5%) respondents expressed a desire to have children in the future with over half (1158) of them wanting to give birth within the next two years.

**Table 1:** Socio-demographic, sexual and fertility characteristics of study participants

***Sociodemographic characteristics (n = 2327)***		
**Characteristic**	**Categories**	**Frequency**, **N (%)**
Age (years)	15–17 years	393 (16.9)
	18–19 years	1934 (83.1)
Country	Kenya	579 (24.9)
	Nigeria	1748 (75.1)
Marital status	Married or living as married	1897 (81.5)
	Single, divorced or separated	430 (18.5)
Currently in school	Yes	376 (16.2)
	No	1951 (83.8)
Religion	Protestant/other Christian	796 (34.2)
	Catholic	124 (5.3)
	Muslim / traditional/ other	1407 (60.5)
***Sexuality and fertility characteristics***		
Sexual frequency	Daily or almost daily	711 (30.6)
	Few times a week	781 (33.6)
	Once a week	163 (7.0)
	Every 2–4 weeks	322 (13.8)
	Half yearly	104 (4.8)
	Don’t know	173 (7.4)
	No response	73 (3.1)
Parity	None	563 (24.2)
	One	1186 (51.0)
	Two	476 (20.5)
	Three or more	102 (4.4)
Desire to have a child^$^	Yes	2174 (93.5)
	No	32 (1.4)
	Don’t know	112 (4.8)
	Can’t get pregnant	8 (0.3)
Timing of desired child*	Less than 1 year	519 (23.8)
	In 1–2 years	639 (29.4)
	> 2 years within 5 years	481 (22.1)
	> 5 years	257 (11.8)
	Don’t know/Refused	281 (12.9)

^$^one participant excluded due to missing data for this variable

*150 participants excluded because they reported no intention to conceive in the future

### Contraception preferences, behaviors and intention

Table 2 illustrates the results on participants’ current desire, current and intended contraceptive behaviors. Overall, 1070 (46.0%) expressed a current desire to use contraception. Slightly over one-third, 866 (37.2%) were currently using contraception. The most reported contraceptive methods used were implants and injectables, followed by male condoms. Almost all current users were satisfied with their contraceptive method. Among respondents who were not currently using contraception, 452 (31%) expressed willingness to use contraception in the following year.

The majority (*n* = 2,012/2,327; 86.5%) of respondents practiced contraceptive behaviors aligning with their stated preferences. Specifically, 75.6% (*n* = 809/1070) of those reporting a desire to use contraception were actively using a method. A greater alignment between contraceptive preference and behavior (*n* = 1,152/1,206; 95.5%) was observed in the sub-group of respondents who did not desire to use contraception. Fifty-one respondents were unsure about their contraceptive preferences and were either using or not using contraception. Notably, 13.5% (*n* = 315/2,327) of respondents exhibited misalignment between their contraceptive behaviors and their stated preferences. This comprised of 54 respondents who did not desire to use contraception but were using it, and 261 respondents with a desire to use contraception who were not using a method.

**Table 2:** Contraception characteristics of sexually-active girls aged 15–19 year in Nigeria and Kenya

Characteristic	Categories	*N* (%)
Desire to use contraception	Yes	1070 (46.0)
	No	1206 (51.8)
	Don’t know	51 (2.2)
Current contraceptive use	Yes	866 (37.2)
	No	1461 (62.8)
Preference-aligned contraceptive use	Yes	2012 (86.5)
	No	315 (13.5)
Currently used contraceptive method*	Male condom	145 (16.7)
	Pills	83 (9.6)
	Emergency pill	12 (1.4)
	Implants	298 (34.4)
	Injectables	227 (26.2)
	IUD	4 (0.5)
	Lactation Amenorrhea Method	21 (2.4)
	Standard method	12 (1.4)
	Other method (safe days, withdrawal)	37 (4.3)
Satisfied with current method^*^	Yes	783 (90.4)
	No	75 (8.7)
	Don’t know / Refused	8 (1.0)
Intend to use contraception in the next one year^$^	Yes	452 (31.0)
	No	811 (55.6)
	Don’t know	196 (13.4)

*Includes only participants who are using a method

^$^Excludes participants currently using a method

### Influence of psychosocial determinants on desire to use contraception

Table 3 illustrates the association between psychosocial determinants and participants’ desire to use contraception. The two psychosocial determinants in the cognitive domain, contraceptive knowledge (aOR = 3.39, 95% CI: 2.02–5.67) and perceived contraceptive relevance (aOR = 3.49, 95% CI: 2.93–4.15) were strongly associated with desire to use contraception in step (1) In step 2, there was a noticeable change in these associations. The association between contraceptive knowledge and desire was lost, but participants with perceived contraceptive relevance were two times more likely to desire to use contraception, albeit this association was weaker than in step 1, (aOR = 1.93, 95%CI: 1.33–2.80). For the emotional domain determinants, higher self-efficacy scores were consistently associated with increased desire to use contraception in both steps, though the association weakened when pooled with the other psychosocial determinants - (aOR = 3.57, 95% CI: 2.50–5.11) for step 1 and aOR = 2.65, 95% CI: 2.29–3.07) for step 2. In both steps, future aspirations scores were not associated with desire to use contraception. The social domain determinants showed weak or no significant associations. Descriptive norms demonstrated a modest association (aOR = 1.33, 95% CI: 1.08–1.65) in step 1, which persisted in step 2 (aOR = 1.26, 95% CI: 1.04–1.52). Perceived reproductive control showed a weak association only in step 2.

**Table 3:** Psychosocial determinants and desire to use contraception

		Step 1*		Step 2**	
	Category	aOR (95% CI)	*p*-value	aOR (95% CI)	*p*-value
**Cognitive domain**					
Contraceptive knowledge	No	Ref		Ref	
	Yes	3.39 (2.02–5.67)	**< 0.001**	2.13 (0.98–4.63)	0.057
Perceived contraceptive relevance	No	Ref		Ref	
	Yes	3.49 (2.93–4.15)	**< 0.001**	1.93 (1.33–2.80)	**0.001**
**Emotional domain**					
Self-efficacy	-	3.57 (2.50–5.10)	**< 0.001**	2.65 (2.29–3.07)	**< 0.001**
Future aspirations	-	1.05 (0.98–1.14)	0.164	0.95 (0.89–1.01)	0.124
**Social domain**					
Descriptive norms	-	1.33 (1.08–1.65)	**0.008**	1.26 (1.04–1.52)	**0.020**
Perceived reproductive control	-	0.99 (0.83–1.17)	0.883	1.06 (1.00-1.12)	**0.036**

* Separate models fitted for each domain of psychosocial determinants adjusting for age, parity, religion, marital status, country and schooling status

** One model fitted including all six psychosocial determinants adjusting for age, parity, religion, marital status, country and schooling status

### Psychosocial determinants and current contraception use

Contraceptive knowledge and perceived contraceptive relevance were consistently associated with current contraceptive use with a slight change of these associations in the presence of other psychosocial determinants (Table 4). A similar pattern was observed regarding the scores on contraceptive self-efficacy and descriptive norms. Contrastingly, scores on future aspirations and perceived reproductive control were not associated with current contraceptive use in both steps.

**Table 4:** Psychosocial determinants and current contraceptive use

Current contraceptive use		Step 1*		Step 2**	
	Categories	aOR (95% CI)	*p*-value	aOR (95% CI)	*p*-value
**Cognitive domain**					
Contraceptive knowledge	No	Ref	-	Ref	-
	Yes	4.14 (3.67–4.67)	**< 0.001**	2.69 (2.38–3.05)	**< 0.001**
Contraceptive relevance	No	Ref	-	Ref	-
	Yes	2.84 (1.74–4.62)	**< 0.001**	1.62 (1.55–1.69)	**< 0.001**
**Emotional domain**					
Self-efficacy	-	3.58 (2.12–6.03)	**< 0.001**	2.70 (1.70–4.29)	**< 0.001**
Future aspirations	-	0.96 (0.63–1.47)	0.858	0.88 (0.51–1.49)	0.625
**Social domain**					
Descriptive norms	-	1.33 (1.07–1.66)	**0.010**	1.28 (1.03–1.60)	**0.026**
Perceived reproductive control	-	0.96 (0.89–1.04)	0.320	1.02 (0.86–1.28)	0.853

* Separate models fitted for each domain of psychosocial determinants adjusting for age, parity, religion, marital status, country and schooling status

** One model fitted including all six psychosocial determinants adjusting for age, parity, religion, marital status, country and schooling status

### Psychosocial determinants and preference-aligned contraceptive use

None of the six psychosocial determinants were associated with preference-aligned contraceptive use except for self-efficacy in step 2 (Table 5).

**Table 5:** Psychosocial determinants and preference-aligned contraceptive use

		Step 1*		Step 2**		
	Categories	aOR (95% CI)	*p*-value	aOR (95% CI)		*p*-value
**Cognitive domain**						
Contraceptive knowledge	No	Ref		Ref		
	Yes	0.73 (0.42–1.28)	0.271	1.01 (0.46–2.19)	0.985	
Perceived relevance	No	Ref		Ref		
	Yes	0.68 (0.20–2.25)	0.525	0.77 (0.33–1.82)	0.555	
**Emotional domain**						
Self-efficacy	-	0.88 (0.59–1.33)	0.549	1.06 (1.05–1.07)	**< 0.001**	
Future aspirations	-	0.76 (0.57–1.03)	0.081	0.80 (0.54–1.18)	0.260	
**Social domain**						
Descriptive norms	-	0.98 (0.87–1.10)	0.705	0.99 (0.95–1.03)	0.613	
Perceived reproductive control	-	1.13 (0.91–1.39)	0.273	1.12 (0.91–1.39)	0.280	

* Separate models fitted for each domain of psychosocial determinants adjusting for age, parity, religion, marital status, country and schooling status

** One model fitted including all six psychosocial determinants adjusting for age, parity, religion, marital status, country and schooling status

We hypothesized that there could be a difference in the mechanisms through which psychosocial determinants influence the alignment between contraceptive desire and use. We explored sub-group analysis replicating the analysis we had performed earlier by fitting two models separately - one for the group of participants who expressed desire to use and another for the group of participants who expressed no desire. In both models, the outcome remained a binary variable parameterized as follows: sub-group 1–1 = desires and currently using; 0 = desires and not currently using and sub-group 2 − 1 = no desire and not currently using; 0 = no desire and currently using. For the sub-group analysis, the 51 participants who responded with a ‘Don’t know’ to the desire question were excluded. The analysis (Table 6) revealed that higher self-efficacy scores were associated with preference-aligned contraceptive use among participants in the group that desired contraceptive use. This effect weakened in the presence of other determinants. Significant associations were observed with contraceptive knowledge and descriptive norms in step 1 but were lost in step 2. No associations were observed with the other determinants. For the analysis involving participants in sub-group 2, we observed a significant negative association between contraceptive knowledge and preference-aligned contraceptive use in step 1 which was diminished in the presence of other determinants.

**Table 6:** Sub-group analysis for psychosocial determinants and preference-aligned contraceptive use

Subgroup 1: (Desire)		Step 1*		Step 2**	
	Categories	aOR (95% CI)	*p*-value	aOR (95% CI)	*p*-value
**Cognitive domain**					
Contraceptive Knowledge	No	Ref		Ref	
	Yes	2.54 (1.48–4.35)	**0.001**	2.00 (0.92–4.35)	0.080
Perceived relevance	No	Ref		Ref	
	Yes	1.37 (0.98–1.91)	0.063	1.16 (0.76–1.79)	0.488
**Emotional domain**					
Self-efficacy	-	2.64 (1.44–4.86)	**0.002**	2.21 (1.02–4.80)	**0.045**
Emotional: Future aspirations	-	0.77 (0.33–1.79)	0.550	0.78 (0.26–2.34)	0.655
**Social domain**					
Descriptive norms	-	1.17 (1.07–1.29)	**0.001**	1.16 (0.99–1.35)	0.058
Perceived control	-	1.02 (0.81–1.28)	0.854	1.01 (0.74–1.39)	0.937
**Subgroup 2: (No desire)**		Step 1*		Step 2**	
	**Categories**	**aOR (95% CI)**	***p*** **-value**	**aOR (95% CI)**	***p*** **-value**
**Cognitive domain**					
Contraceptive Knowledge	No	Ref		Ref	
	Yes	0.19 (0.04–0.86)	**0.030**	0.35 (0.09–1.34)	0.124
Perceived relevance	No				
	Yes	0.65 (0.06–7.52)	0.732	0.83 (0.12–5.96)	0.852
**Emotional domain**					
Self-efficacy	-	0.63 (0.28–1.44)	0.277	0.83 (0.62–1.09)	0.176
Future aspirations	-	0.68 (0.36–1.30)	0.244	0.86 (0.53–1.39)	0.531
**Social domain**					
Descriptive norms	-	0.84 (0.64–1.10)	0.200	0.86 (0.69–1.07)	0.174
Perceived reproductive control	-	1.74 (0.98–3.10)	0.060	1.70 (0.98–2.97)	0.060

* Separate models fitted for each domain of psychosocial determinants adjusting for age, parity, religion, marital status, country and schooling status

** One model fitted including all six psychosocial determinants adjusting for age, parity, religion, marital status, country and schooling status

## Discussion

This study employed a theory-driven approach to examine the association between six psychosocial determinants and three outcomes on the contraceptive use journey among sexually-active girls aged 15–19 years in Kenya and Nigeria, blending the ideation theory and socio-ecological model in the analytic framework. Notably, slightly over half of the sexually-active girls expressed no desire to use contraception and two thirds were not currently using a method affirming the high unmet contraception need demonstrated through findings from recent studies [8]. These two indicators are important considering that the study was conducted within the context of an active project, of which the goal is to increase demand for and voluntary uptake of modern contraception. These findings implicate an enormous opportunity for the project to demonstrate impact. An important consideration for ASRH programs in the context where this study was based is that motherhood is a valued achievement and adolescent girls, especially those married, experience substantive pressure to prove their fertility [33]. Programs in these settings should invest in designing demand creation components that build a strong value proposition for contraception and increase girl’s agency to circumvent these societal expectations [34]. Shifting girls’ mindsets to postpone pregnancy immediately after marriage would culminate in increased desire and demand for contraception which would delay childbearing, avert the consequences of early pregnancy and provide girls adequate opportunities to attain their full life potential. To shift mindsets may take a long time, so in the short-term implementers of ASRH programs should remain conscious of the highly desired fertility and respect the reproductive preferences and decisions made by their target beneficiaries but profile population segments who could be impacted by demand generation, expanded method access and support to consistently use contraception [35]. The high alignment between desire for contraception and the use status was comparable to studies by Bullington et al. and Rothschild et al. and implies that most sexually-active girls were exercising choices based on their self-defined preferences [20, 35]. As argued by these authors, ASRH programs should vacate the assumption that all sexually-active or married adolescent girls are at risk for pregnancy and need contraception. Instead, programs could employ preference-aligned contraceptive use as a putative measure of implementation success by determining the program’s capacity to foster person and rights-centered contraceptive delivery mechanisms. That notwithstanding, adequate investments are needed to address the documented lack of desire, which could be addressed by increasing contraceptive knowledge, addressing myths, misconceptions and socio-cultural norms, enhancing convenient access to high quality counselling as well as ensuring consistent availability of a diverse menu of contraceptive methods [34].

Our study is among few studies that have specifically focused on girl’s desire to use contraception. Desire is a psychological construct that captures what individuals prefer to do at a given point in time. Desire is different from intention - which relates to demanded or planned behavior - and differs from current use, which reflects actual behavior. In our study, we observed that desire to use contraception was associated with a perception of the relevancy of contraceptives towards pursuing one’s self-defined goals. This finding concurs with earlier research suggesting that an individual’s desire towards a certain behavior drives their interest for the product or service - a proven prerequisite for initiating action to get it [36]. The perception that contraception is relevant or beneficial in one’s life fosters desire to use it. We postulate that programs that impart comprehensive contraception knowledge do contribute to desire, but knowledge alone is inadequate to influence desire. Apart from knowledge, as demonstrated in our study, persuasive evidence on the role of contraceptive self-efficacy in driving desire, intention to use and actual use of contraception is emerging [37, 38]. We postulate that once girls gain confidence in their ability to decide, access and use contraception, they become more optimistic about achieving a successful outcome and therefore develop a desire for it. Similarly, we observed a positive association between high scores on descriptive norms and desire, affirming existing literature that when young women believe that contraception is culturally acceptable and normalized, their desire to use increases [39]. It is surprising that perceived reproductive control by others did not influence desire for contraceptive use. We hypothesize that girls in the study’s context define their preferences autonomously but will adopt shared decision making when accessing and using contraception. This is an area ASRH programs can leverage to circumvent pervasive societal norms.

Regarding current contraceptive use, our study established a consistent interplay of the psychosocial determinants across the three ideation domains. We established that adolescent girls with adequate knowledge, who perceived contraceptives to be relevant and with higher scores on self-efficacy and descriptive norms were more likely to be using a contraceptive method. The findings underscore the importance of designing interventions that address the cognitive and emotional competencies related to contraceptive use as a foundation for accelerating eligible candidates through the contraceptive adoption pathway, as part of implementing high impact family planning practices [40]. The slight shifts in the associations between the determinants in the models when determinants were pooled together concur with the argument that components of the various domains in the ideation model function synergistically to influence the processing of information when making decisions towards the adoption of constructive behaviors [41]. Similar observations were made in a recent study on psychological determinants and intention to use hormonal contraceptives among adolescent girls in Ghana [42]. We suspect that ongoing efforts to increase contraceptive autonomy and agency among adolescent girls in these contexts are progressively contributing to improved autonomous decision-making and challenging existing deeply entrenched gender and power disparities.

We observed that no psychosocial determinants showed an association with preference-aligned contraceptive use. However, in the sub-analysis involving participants with desire to use contraception, higher self-efficacy scores were associated with preference-aligned contraceptive use affirming self-efficacy’s indisputable influence on the contraceptive uptake pathway from desire, through intention and in the use [12]. The lack of associations is surprising because logic dictates that psychosocial determinants would show positive associations with person-centered contraceptive behaviors. This finding should not spell pessimism because person-centered contraceptive measurement is a rapidly emerging space, and the sexual and reproductive health (SRH) sector is yet to evolve with consensus on which are the most robust measures. Moreover, recent studies that evaluated the alignment of contraceptive use and desire suggest that more research is warranted to gain a deeper understanding of these newer metrics, recommend cognitive interviewing techniques to refine the framing of the questions used and to explore the intermediate factors that could be modified to optimize their attainment [35]. Besides, using this measure has illuminated two distinct categories of participants who need support in unique ways. Girls with no desire to use contraception who also were not currently using it will benefit from components that emphasize creating a strong value proposition for contraception while those with a desire who were not currently using will benefit from programs that focus on increasing decision-making agency and improving access [35]. This differentiated support grounded in girls’ preferences could be more effective than providing a one-size-fits-all intervention to sexually-active girls and is worth exploration through implementation research.

### Limitations

This study is not without its drawbacks. First, our findings are not generalizable to all adolescent girls 15–19 years in the two contexts. Due to consenting requirements in Kenya, unemancipated minors (15–17) were excluded. In Nigeria, unmarried girls were also not recruited for the study. Furthermore due to consenting requirements, our study mainly consisted of married adolescent girls, or those who have a prior birth and does not represent the many nulliparous and unmarried girls who are also reached by the A360 project. This is regrettable because this group has unique attributes which expose them to a disproportionately higher risk for unintended and early pregnancies. Second, we employed self-reports to obtain data for our independent and dependent variables. Self-reports are subject to socially desirable reporting during data collection. To mitigate these biases, we implemented comprehensive training on rapport building by enumerators, used same sex interviewers and observed visual and auditory privacy during data collection. Third, psychosocial determinants were measured using tools adapted from previous validation studies. Not all the tools were validated in our study geographies which could affect the validity of the measures. To mitigate this risk, we employed some adaptations to the validated questions to suit our study’s contexts, used local language translations and performed internal consistency checks before the analysis. Despite these limitations, we believe that our study has contributed valuable evidence to advance the SRH sector.

## Conclusions

Our results show that individual-level factors, such as contraceptive knowledge, perceived contraceptive relevance and self-efficacy, are potential modifiable determinants for both desire to use contraception and actual contraceptive use, even in the presence of social influences like descriptive norms. SRH programs should therefore prioritize helping girls understand how contraception can support their future life goals. Specifically, programs should first ensure that girls have accurate contraceptive knowledge, as this might be essential before girls even start wanting or using contraception. However, focusing on these psychosocial factors alone might be insufficient in achieving alignment between girls self-defined preferences and their contraceptive behaviors. Future research should explore additional modifiable factors, such as contraceptive autonomy that may affect how girls translate their contraceptive desires into actual behavior.

## Electronic Supplementary Material

Below is the link to the electronic supplementary material.

Supplementary Material 1

Supplementary Material 2

## Data Availability

Data supporting the findings of this study are available on this link - 10.6084/m9.figshare.28682957.v1.
