# Supplementary material for: Psychosocial determinants of contraceptive desire and use among sexually-active adolescent girls in Kenya and Nigeria: implications for girl-centered contraceptive programs
Source: Contracept Reprod Med. 2025 Dec 8;11:6. doi: 10.1186/s40834-025-00416-w (PMC12797765; doi:10.1186/s40834-025-00416-w)
Supplement: Supplementary file 1 — Supplementary Material 1 [file 40834_2025_416_MOESM1_ESM.docx]

Supplementary Material 1

**Table 7: Summary description of measures used for assessing the psychosocial determinants**

| **Determinant** | **Nature of scale** | **Questions** | **Response options** | **Variable definition** |
| --- | --- | --- | --- | --- |
| Contraceptive knowledge | Composite of three questions adapted from Nsubuga et al.,[29] | 1. *Have you ever heard about child spacing/family planning/contraceptive method?* 2. *Which method(s) have you heard about?* 3. *Do you know of a place where or person from whom you would feel comfortable getting services and products to delay or avoid getting pregnant?* | *1 = Yes*  *0 = No* | Contraceptive knowledge was present if the participant had heard about contraceptive methods, mentioned at least one valid method and stated a valid location or person from where contraceptive services and products could be obtained |
| Perceived relevance of contraception | Composite of responses to two statements and one question | 1. *Using contraceptive can allow a girl to complete her education, find a better job and have a better life* 2. *Using contraceptive can allow a girl to achieve her life goals* 3. *What do you think are the benefits of family planning*? | For the two statements  *Disagree = 1*  *Neutral = 2*  *Agree = 3*  For the question  *1 = Yes*  *0 = No* | Contraception was perceived to be relevant if a participant agreed with the two statements and stated a valid benefit of using contraception |
| Contraceptive self-efficacy | Nine-item Likert scale adapted from Whiting-Collins et al., [30] | Statements measured participant’s confidence to negotiate or discuss contraception with their sexual partners, friends and providers and to access and use contraception | Five response options  *Strongly disagree* = *1*  *Disagree* = *2*  *Undecided* = *3*  *Agree = 4*  *Strongly agree = 5* | The sum of the scores from all items divided by number of items to generate average item scores - higher scores depicting greater self-efficacy |
| Future aspirations | Four-item scale adapted from Krug et al.,[31] | Statements assessed participants’ awareness of their life goals, confidence in their ability to pursue the goals and belief in the value of contraception towards attaining the goals | Same as self-efficacy | Generated cumulative scores for the four items - higher scores depict greater confidence to pursue their future aspirations |
| Perceived reproductive control | Five-item Likert scale adapted from Rowlands & Walker [32] | Statements assessing participants’ perception of the influence of the husband, parents, in-laws or fate in making reproductive health decisions | Same as self-efficacy | Same as self-efficacy - higher scores depicts a stronger perception of reproductive control by others |
| Descriptive norms | Three-item Likert scale adapted from Krug et al., [31] | Statements assessed participants’ estimation of the proportion of adolescent girls from their communities who use contraception. | Four response options  *Most of them = 2*  *Less than half of them = 1*  *None of them/don’t know = 0* | Scores were cumulated to generate a sum score ranging between 0 and 6. Higher scores indicate better descriptive norms. |
