# Supplementary material for: Psychosocial determinants of contraceptive desire and use among sexually-active adolescent girls in Kenya and Nigeria: implications for girl-centered contraceptive programs
Source: Contracept Reprod Med. 2025 Dec 8;11:6. doi: 10.1186/s40834-025-00416-w (PMC12797765; doi:10.1186/s40834-025-00416-w)
Supplement: Supplementary file 2 — Supplementary Material 2 [file 40834_2025_416_MOESM2_ESM.docx]

Supplementary Material 2

**Table 8: Socio-psychological determinants: prevalence, mean scores and internal consistency**

| Cognitive factors | Categories | N | % | No. of items |  |
| --- | --- | --- | --- | --- | --- |
| Good knowledge | Yes | 1852 | 79.6 | 3 |  |
|  | No | 475 | 20.4 |  |  |
| Perceived relevance | Yes | 1686 | 72.5 | 3 |  |
|  | No | 641 | 27.6 |  |  |
|  |  |  |  |  |  |
| Emotional and social factors | **Mean** | **SD** | **IQR** | **No. of items** | **Cronbach’s alpha** |
| Perceived self-efficacy | 3.89 | 0.82 | 0.89 | 9 | 0.93 |
| Future aspirations | 4.04 | 0.65 | 0.75 | 4 | 0.74 |
| Perceived reproductive control | 3.06 | 0.81 | 1.17 | 6 | 0.72 |
| Descriptive norms | 3.35 | 1.79 | 3.00 | 3 | 0.72 |
